# Characterization of the CDAA Diet-Induced Non-alcoholic Steatohepatitis Model: Sex-Specific Differences in Inflammation, Fibrosis, and Cholesterol Metabolism in Middle-Aged Mice

**DOI:** 10.3389/fphys.2021.609465

**Published:** 2021-02-22

**Authors:** Dániel Kucsera, Viktória E. Tóth, Dorottya Gergő, Imre Vörös, Zsófia Onódi, Anikó Görbe, Péter Ferdinandy, Zoltán V. Varga

**Affiliations:** ^1^Department of Pharmacology and Pharmacotherapy, Semmelweis University, Budapest, Hungary; ^2^Hungarian Centre of Excellence for Molecular Medicine – Semmelweis University (HCEMM-SU) Cardiometabolic Immunology Research Group, Budapest, Hungary; ^3^Pharmahungary Group, Szeged, Hungary

**Keywords:** cirrhosis, liver failure, cholesterol, lipoprotein, heart, metabolic syndrome, NAFLD, menopause

## Abstract

**Background:**

The prevalence of non-alcoholic steatohepatitis (NASH) rapidly increases with associated metabolic disorders such as dyslipidemia; therefore, NASH is now considered an independent risk factor of cardiovascular diseases. NASH displays sex-linked epidemiological, phenotypical, and molecular differences; however, little is known about the background of these sex-specific differences on the molecular level.

**Objectives:**

We aimed to assess sex-specific differences in the expression of inflammatory and fibrotic genes, as well as in cholesterol metabolism, focusing on the expression of *Pcsk9* in several tissues in a mouse model of NASH that shows the typical features of the human condition.

**Methods and Results:**

We fed 10-months-old male and female C57Bl/6J mice with a NASH-inducing CDAA or corresponding control diet for 8 weeks. We found that, compared to the control male mice baseline, hepatic *Pcsk9* expression as well as serum PCSK9 level was significantly higher in females, and both circulating PCSK9 level and the hepatic Pcsk9 gene were markedly decreased in female mice during NASH development. Histological analysis revealed that male and female mice develop a similar degree of steatosis; however, fibrosis was more pronounced in males upon CDAA diet feeding. Strikingly, female mice have higher hepatic expression of the pro-inflammatory cytokines (*Il1b, Ifng*), and increased IL-1β cleavage by the NLRP3 inflammasome, and a decrease in Clec4f^+^ resident Kupffer cell population in comparison to males in the CDAA-fed groups.

**Conclusion:**

This is the first demonstration that there are critical sex-specific differences during NASH development in middle-aged mice regarding inflammation, fibrosis, and cholesterol metabolism and that changes in PCSK9 and IL-1β are likely important contributors to sex-specific changes during the transition to NASH.

## Introduction

Non-alcoholic fatty liver diseases (NAFLD) and subsequent non-alcoholic steatohepatitis (NASH) are becoming the most significant causes of liver disease worldwide, without any approved drugs for their treatment. Obesity, as the primary cause of these progressive liver diseases, initially induces simple steatosis. Later the extensive lipid and cholesterol accumulation in hepatocytes and in Kupffer cells along with damage to intestinal barrier function leads to hepatocyte necrosis triggering Kupffer cell and hepatic stellate cell activation leading to the progression of NASH to cirrhosis. NAFLD and NASH are associated with a pro-atherogenic lipid profile; therefore, both conditions are now considered important cardiovascular risk factors. Evidence from epidemiological as well as from animal studies suggest that the incidence of NAFLD is higher in males compared to premenopausal female patients (Lazo et al., 2013; Ballestri et al., 2017). However, the risk of NASH and advanced fibrosis is indeed higher in postmenopausal females than males independently of metabolic factors (Bambha et al., 2012). Premenopausal women have lower rates of NAFLD compared with men; however, this protection is lost following perimenopause when NAFLD prevalence becomes similar or even higher in comparison to age-matched male counterparts (DiStefano, 2020). The pathophysiology of NASH and NAFLD displays sex-linked differences, explaining that liver-targeted drugs may target distinct mechanisms in men and women. This might be the case in a randomized phase II clinical trial evaluating the therapeutic effect of cenicriviroc (a dual C-C chemokine receptor 2 and 5 antagonist) against NASH with fibrosis. Based on the results, the drug had a positive effect in men, but not in women, on the improvement of fibrosis after the 1 year follow-up period (Friedman et al., 2018). However, the molecular mechanism behind these clinical observation is unclear due to the limited number of preclinical studies, investigating sex- and age-specific changes occurring in NASH models.

Cholesterol and lipid metabolism shows major sex-specific differences. This sexual dimorphism is a result of a complex network of sex hormone actions in combination with other, possibly sex-specific, direct or indirect modulators of lipid metabolism (e.g., differences in insulin and adipokine action) (Wang et al., 2011). Proprotein convertase subtilisin kexin type 9 (PCSK9) is a critical regulator of cholesterol metabolism, primarily by inhibiting low-density lipoprotein receptor (Ldlr) recycling, and thereby blocking the cellular uptake of low density lipoprotein-cholesterol (LDL-C) (Horton et al., 2007). The discovery of the marked effect of PCSK9 on LDL-C serum level has led to the rapid development of PCSK9 inhibitors for the treatment of hypercholesterolemia to reduce the risk of subsequent cardiovascular disease (CVD) development (Dullaart, 2017). A marked reduction in major cardiovascular events have been reported in clinical trials assessing the efficacy of PCSK9 monoclonal antibodies (Sabatine et al., 2017), as well as PCSK9 inhibiting small interfering RNAs (Ray et al., 2017), with no specific safety concerns reported to date. A limited number of studies have so far explored whether PCSK9 shows any sex-specific differences in conditions like myocardial infarction (Zhang et al., 2019), or hypercholesterolemia (Roubtsova et al., 2015). Nevertheless, these changes might be important in understanding sex-specific differences in response to PCSK9 inhibitor therapy (Sacks et al., 2018; Ferri et al., 2020).

It has been shown that besides extracellular circulating PCSK9, both intracellular, and extrahepatic PCSK9 expression and other PCSK9-mediated pathways are also involved in regulating cholesterol metabolism (Tang et al., 2020). In addition, PCSK9 has a central role in macrophage cholesterol efflux regulation (Adorni et al., 2017), and thereby it affects pro-inflammatory cytokine production and inflammatory mechanisms (Tang et al., 2017). Interestingly, inflammation as a result of endotoxin challenge or NLRP3 inflammasome activation seems to be a robust activator of PCSK9 expression and secretion (Feingold et al., 2008; Ding et al., 2020). Recognition of the complex effects of PCSK9 are leading toward a broader use of PCSK9 inhibitors in diseases such as NAFLD and NASH (Ajam et al., 2017; Dimakopoulou et al., 2018).

Based on these studies, we aimed to assess sex-specific differences in inflammation, fibrosis, and cholesterol metabolism, including PCSK9 expression across different tissues in a non-obese and non-alcoholic model of NASH in middle-aged C57Bl/6J mice, which might better resemble the human condition that occurs in the middle-aged and elderly population.

## Materials and Methods

### Experimental Animals and Diets

Adult female and male C57Bl/6J mice were purchased from the Oncological Research Center, Department of Experimental Pharmacology, Budapest, Hungary. Mice were maintained under a 12-12 light-dark cycle. Cages were occupied by 2–4 mice per cage. Diet and tap water were available *ad libitum* throughout the experiment. The investigation conforms to the Guide for the Care and Use of Laboratory Animals published by the US National Institutes of Health (NIH publication No. 85-23, revised 1996), to the EU Directive (2010/63/EU), and was approved by the National Scientific Ethical Committee on Animal Experimentation (PE/EA/1912-7/2017, Budapest, Hungary). The Control (E 15668-04) and Choline Deficient L-Amino Acid defined (CDAA) diet (E 15666-94) was purchased from SSNIFF GmbH (Soest, Germany).

### Non-alcoholic Steatohepatitis Model

**FIGURE 1:**
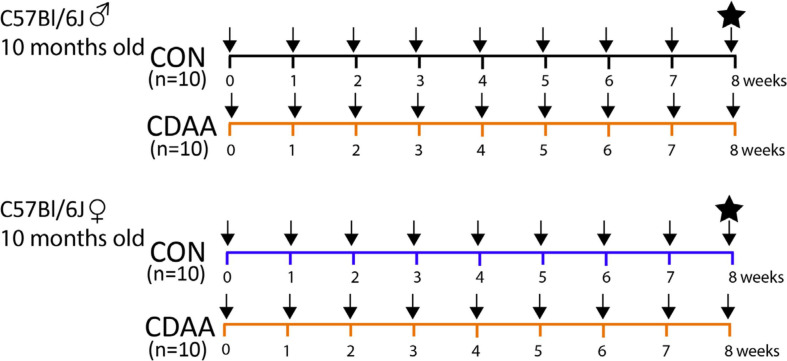
Experimental protocol. Ten-months-old male and female C57Bl/6J mice (*n* = 10) were fed with CDAA or CON diet for 8 weeks. Black arrows indicate weekly body weight measurements. The black star indicates the termination of mice in the 9th week, when blood and organ samples were collected. CDAA, Choline Deficient L- Amino acid-defined diet; CON, control diet.

Twenty female and twenty male C57Bl/6J mice at the age of 10 months (i.e., considered perimenopausal age in female mice) (Diaz Brinton, 2012), weighing on average 30 g were randomly assigned to the control diet-fed group (CON, *n* = 10) or CDAA diet-fed group (CDAA, *n* = 10). Body weight was measured weekly. After 8 weeks of diet, the mice were sacrificed, and their internal organs were stored for histological and molecular analyses (Figure 1). Blood, collected during animal termination, was left at room temperature for 15 min to allow coagulation, then it was centrifuged at 2,500 G at 4°C for 15 min. Serum was collected and snap frozen in liquid nitrogen for further analyses.

### Histologic Analysis

Liver tissues were fixed in neutral buffered formalin for 24 h, then dehydrated and embedded in paraffin. Five μm thick sections were used for histological analyses and immunohistochemistry. All the staining was visualized and images were captured with Leica LMD6 microscope (Wetzlar, Germany).

#### Hematoxylin and Eosin Staining

Paraffin embedded liver sections were stained with hematoxylin and counterstained with eosin after initial deparaffinization and hydration for evaluation of hepatic morphologic and pathologic alterations.

#### Sirius-Red Staining

Liver sections after initial preparations (see above) were stained with 0.0125% picrosirius red for 1 h, then washed with 1% acetic acid. The extent of hepatic fibrosis was analyzed and quantified by the ImageJ software.

#### Immunohistochemistry

The properly prepared (see above) liver sections underwent antigen retrieval (citrate buffer pH = 6 or Tris buffer pH = 9 at 95°C) for 15 min. Endogenous peroxidase activity was blocked by 3% H_2_O_2_ in PBS solution, then the sections were blocked with 2.5% goat serum or 2.5% horse serum in PBS and 2% milk powder or bovine serum albumin. Primary antibodies—Iba1 for macrophages (019-19741, Wako Pure Chemical Industries, Osaka, Japan), Clec4/Clecsf13 for Kupffer cells (MAB2784, R&D Systems, Minneapolis, MN, United States), MPO for neutrophil granulocytes (AF3667, R&D systems, Minneapolis, MN, United States), and CD3e for T cells (D7A6E, Cell Signaling Technology, Danvers, MA, United States)—were diluted (1:2,500, 1:200, 1:100, 1:200, respectively) in goat serum or horse serum and were incubated overnight in blocking solutions at 4°C. After the sections were washed three times with PBS, the sections were incubated with anti-rabbit IgG (7074S, Cell Signaling Technology, Leiden, The Netherlands), anti-goat IgG (MP-7405, Immpress reagents, Vector Laboratories, Burlingame, CA, United States), or anti-rat IgG (MP-7444, Vector Laboratories, Burlingame, CA, United States) conjugated with a peroxidase. Secondary antibodies were washed three times with PBS and signals was developed with diaminobenzidine (ImmPACT DAB EqV Peroxidase (HRP) Substrate, Vector Laboratories, Burlingame, CA, United States).

### RNA Isolation, cDNA Synthesis, and qRT-PCR

Total RNA was isolated from liver, heart, kidney, small intestine, and adrenal samples (*n* = 6/group) with the isopropanol/chloroform precipitation method. Samples were homogenized in QIAzol (Qiagen, Netherlands). Homogenates were centrifuged, DNA and proteins were separated with chloroform. Total RNA was precipitated with isopropanol and pellets were washed four times with ethanol (vWR, PA, United States). RNA was resuspended with nuclease free water, and RNA concentration was measured with a Nanophotometer (Implen GmbH, Munich, Germany).

Reverse transcription was performed from 1 μg of total RNA with a Sensifast cDNA synthesis kit (Bioline, London, United Kingdom) according to the manufacturer’s protocol. The acquired cDNA was diluted 20x with RNase free water. Target genes were amplified using a LightCycler^®^ 480 II instrument (Roche, Germany) using the SensiFAST SYBR Green master mix (Bioline, United Kingdom). Polymerase was heat activated for 2 min at 95°C, target genes were amplified for 40 cycles. Results were calculated with the 2^–ΔΔCp^ evaluation method. Primer sequences are available in Supplementary Table 1.

### Western Blot

Frozen liver samples were homogenized in RIPA lysis buffer (20 mM of Tris-HCl, 150 mM of NaCl, 1% NP-40). Protein concentration was measured by the bicinchoninic acid method using bovine serum albumin (BSA) as standard (Thermo Fisher Scientific, Rockford, IL, United States). Twelve μg of protein was loaded onto 4–20% polyacrylamide gel. After separation by gel electrophoresis, proteins were transferred (Criterion Blotter, BioRad, Hercules, CA, United States) onto PVDF membranes (BioRad, Hercules, CA, United States). After successful transfer, the membrane was blocked with 5% BSA (Sigma-Aldrich, St. Louis, MO, United States) solution for 1 h at room temperature. Afterward, the membrane was incubated overnight at 4°C with a primary antibody (dissolved in 5% BSA solution) against IL-1β (ab9722, Abcam, Cambridge, MA, United Kingdom, 1:1,000), NLRC4 (D5Y8E, Cell Signaling Technology, Danvers, MA, United States, 1:2,500 dilution), and NLRP3 (D4D8T, Cell Signaling Technology, Danvers, MA, United States, 1:2,500 dilution), followed by washing with 0.05% Tris-buffered saline with Tween 20 (TBS-T) (3 × 10 min). The membrane was incubated with a secondary antibody (horseradish peroxidase-conjugated goat anti-rabbit, 7074, Cell Signaling Technology, Danvers, MA, United States, 1:5,000 dilution) dissolved in 5% BSA solution for 2 h at room temperature, followed by 3 × 10 min wash. For band detection, the membranes were incubated with enhanced chemiluminescence reagent (Clarity Max Western, BioRad, Hercules, CA, United States) for 5 min and the signal was recorded with the ChemiDoc XRS + System (BioRad, Hercules, CA, United States). Band intensity was evaluated using the Image Lab Software (BioRad, Hercules, CA, United States). Loading control was determined by measuring GAPDH content. After stripping the membrane, it was incubated overnight at 4 °C with a primary antibody against GAPDH (D16H11, Cell Signaling Technology, Danvers, MA, United States, 1:5,000). After washing, the stripped membrane was incubated with a secondary antibody (horseradish peroxidase-conjugated goat anti-rabbit 1:5,000 dilution), followed by washing with TBS.

### ELISA

Circulating PCSK9 level was measured from frozen serum samples with a Mouse PCSK9 ELISA kit purchased from Mybiosource (San Diego, CA, United States). A hundred μL of blank, standards, and samples were loaded into the wells and were incubated at room temperature for 90 min with gentle shaking (100 rpm). After 3 × 1 min washing with Wash Buffer Working Solution (dilution 1:20 with distilled water), 100 μL of Biotin-Labeled Detection Antibody Working Solution (dilution 1:100 with Detection Antibody Diluent) was loaded into the wells and was incubated at 37°C for 1 h. After 3 × 1 min washing with the Wash Buffer Working Solution, 100 μL of Streptavidin-HRP Working Solution (dilution 1:100 with Streptavidin-HRP Diluent) was added into the wells and was incubated at 37°C for 45 min. The plate was washed 5 × 1 min with the Wash Buffer Working Solution, then 100 μL of TMB Substrate Solution was added and incubated at 37°C for 30 min. Afterward, 100 μL of Stop Solution was added. The colorimetric reaction was measured at 450 nm with a Thermofisher Multiskan Go spectrophotometer (Waltham, MA, United States).

### Determination of Serum Triglyceride and Total Cholesterol Content

Triglyceride and total cholesterol content were measured from serum using a colorimetric method (Diagnosticum, Budapest, Hungary). One micro liter of serum was added to 100 μL of reagent, and after gentle shaking and 5 min of incubation at 37°C, the absorbance values were measured at 505 nm with a Thermofisher Multiskan Go spectrophotometer (Waltham, MA, United States).

### Data and Statistical Analysis

All values are presented as mean ± standard error of mean (SEM). The statistical analysis was performed with the GraphPad Prism software. Two-way ANOVA followed by Tukey’s *post hoc* test was used for multiple comparisons. ^∗^*P* < 0.05 was considered significant, ^∗∗^*P* < 0.01 was considered significant, ^∗∗∗^*P* < 0.001 was considered significant.

## Results

### CDAA Diet Leads to Macroscopic and Microscopic Steatohepatitis

**FIGURE 2:**
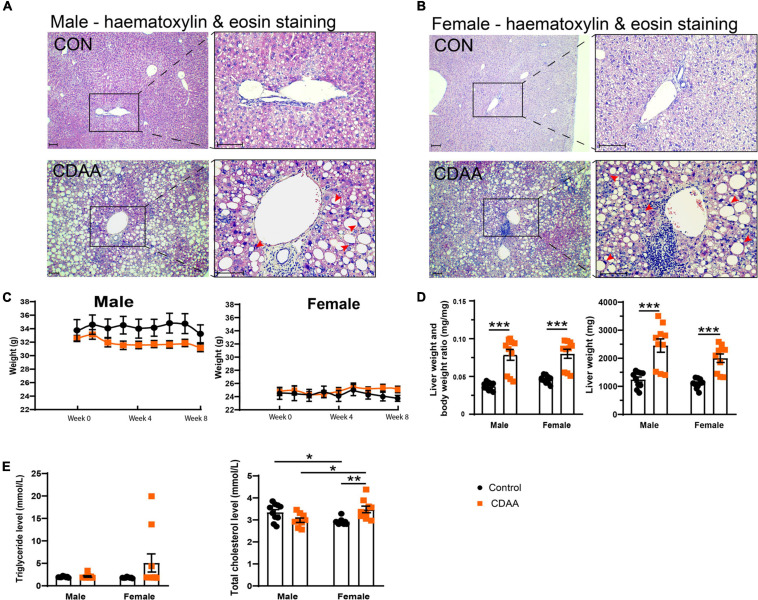
Hepatic injury in the mouse CDAA NASH model. Histological assessment of hepatic pathological alterations on hematoxylin-eosin stained sections in female and male mice **(A,B)**. Weekly body weight alterations of male and female mice, respectively **(C)**. Assessment of liver enlargement by liver weight and liver weight to body weight ratio (mg⋅mg-1) **(D)**. Serum triglyceride and total cholesterol levels **(E)**. Scale bar indicates 100 μm. Results are shown as mean ± standard error of mean (SEM). *P < 0.05 was considered significant, **P < 0.01 was considered significant, ***P < 0.001 was considered significant.

Eight weeks of the CDAA diet in 10-month-old male and female mice led to the typical morphological features of steatohepatitis (Figure 2). Hematoxylin and eosin staining of the livers from male and female mice, respectively, showed macrovesicular steatosis in both groups with eccentric, displaced hepatocyte nuclei (Figures 2A,B). There were several areas with apparent inflammatory infiltrates. Eight weeks of the CDAA diet did not lead to significant differences in body weight (Figure 2C), making this model an excellent tool to investigate the direct effect of NASH without the confounding effects of body weight gain. Analysis of the liver weight, and liver weight to body weight ratio showed increased liver weights in both sexes (Figure 2D), further supporting the development of steatohepatitis. There were no significant changes in serum triglyceride levels due to the CDAA diet. Baseline serum total cholesterol was lower in female mice. Upon CDAA feeding there was a sex-specific difference, i.e., females showed a significant increase in cholesterol levels.

### Expression of *Pcsk9*, *Ldlr*, and *Cd36* in Liver, Heart, Small Intestine, and Adrenals

**FIGURE 3:**
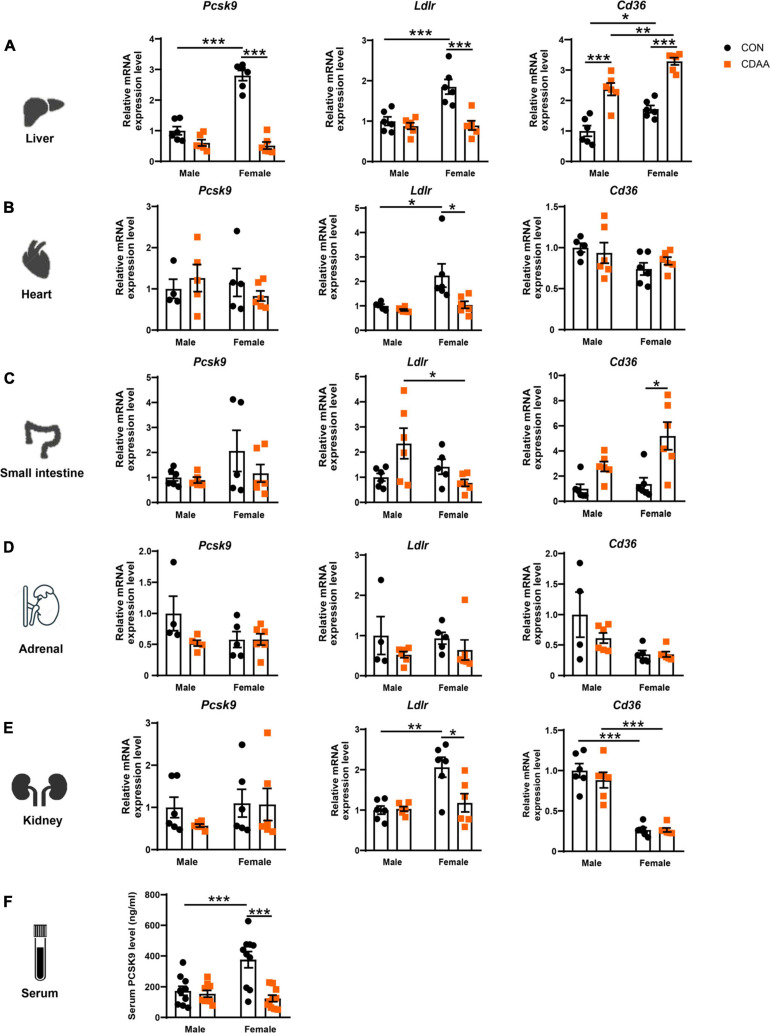
Changes of expression *Pcsk9, Ldlr*, and *Cd36* genes upon CDAA-induced NASH in the liver, heart, small intestine, adrenal, and kidney, as well as PCSK9 serum concentration. Relative gene expression of *Pcsk9, Ldlr*, and *Cd36* from liver **(A)**, heart **(B)**, small intestine **(C)**, adrenal gland **(D)**, and kidney **(E)** was measured by qRT-PCR and was normalized to the *Rlp13a* gene. Serum PCSK9 concentration **(F)**. Results are shown as mean ± standard error of mean (SEM). *P < 0.05 was considered significant, **P < 0.01 was considered significant, ***P < 0.001 was considered significant.

The *Pcsk9* gene is primarily expressed in the liver, though a smaller extent of expression has been also reported in the small intestine, kidney, adrenal gland, and in the myocardium. Accordingly, circulating PCSK9 levels are mostly reflected by the gene expression changes seen in the liver (Peterson et al., 2008). Therefore, *Pcsk9*, *Ldlr* and *Cd36* expression has been determined in all these organs (Figures 3A–E). We found a higher baseline gene expression of *Pcsk9* in the liver of female mice, that was significantly reduced in the CDAA diet-fed animals (**A**). We also found higher baseline expression of *Ldlr* in female mice in the liver, heart, and ileum, which was reduced due to the CDAA diet (Figures 3A–C). Serum PCSK9 concentration showed a similar pattern to hepatic gene expression. Female mice had an increased baseline level in circulating PCSK9 that was significantly reduced upon CDAA feeding (Figure 3F).

CD36 is a well-known driver of liver steatosis and injury (Koonen et al., 2007). CD36 is also under the control of PCSK9 similarly to LDLR (Demers et al., 2015). We found an induction of the hepatic *Cd36* gene in both males and females upon CDAA feeding. Interestingly, female mice had higher baseline expression of hepatic *Cd36*, and its induction was significantly higher in female mice fed CDAA, than in male counterparts (Figure 3A). A similar trend was observed in regards to *Cd36* expression in the small intestine (Figure 3C). Interestingly, the *Ldlr* expression of the kidney showed a similar pattern to the liver and heart, whereas *Cd36* markedly reduced in the kidneys of female mice, irrespective of the diet used (Figure 3E).

### Differences in Fibrosis Development in Male and Female CDAA Diet-Fed Mice

**FIGURE 4:**
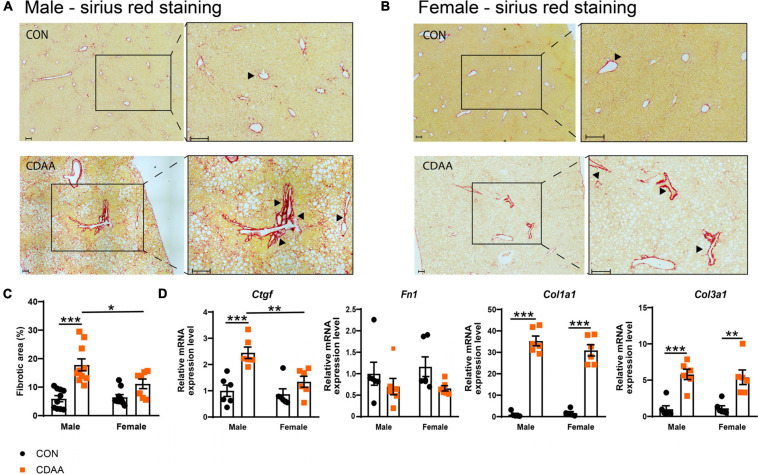
Hepatic fibrosis in CDAA-induced NASH. Determination of the extent of liver fibrosis by histological assessment of hepatic pathological alterations on picrosirius red-stained liver sections in males **(A)** and females **(B)**, and by the extent of fibrotic area (%) **(C)**. qRT-PCR was performed to assess relative expression of the indicated fibrotic genes and it was normalized to the *Rpl13*a gene **(D)**. Black arrows indicate fibrotic areas. Scale bar indicates 100 μm. Results are shown as mean ± standard error of mean (SEM). *P < 0.05 was considered significant, **P < 0.01 was considered significant, ***P < 0.001 was considered significant.

Fibrotic changes and subsequent cirrhosis development are considered to be the end-stage of steatohepatitis. In our diet-induced NASH model, we found more pronounced fibrosis in male mice with the quantitative analysis of the picrosirius red staining (Figures 4A–C). A similar pattern was detected in the expression of the connective tissue growth factor *(Ctgf)* gene (Figure 4D). A similar degree of fibrotic changes were detectable on collagen mRNAs (*Col1a1* and *Col3a1*) (Figure 4D). In the case of the fibronectin *(Fn1)* gene, we could not find any significant difference among the groups tested.

### Certain Inflammatory Changes Are More Pronounced in Female Mice Upon CDAA Diet Feeding

**FIGURE 5:**
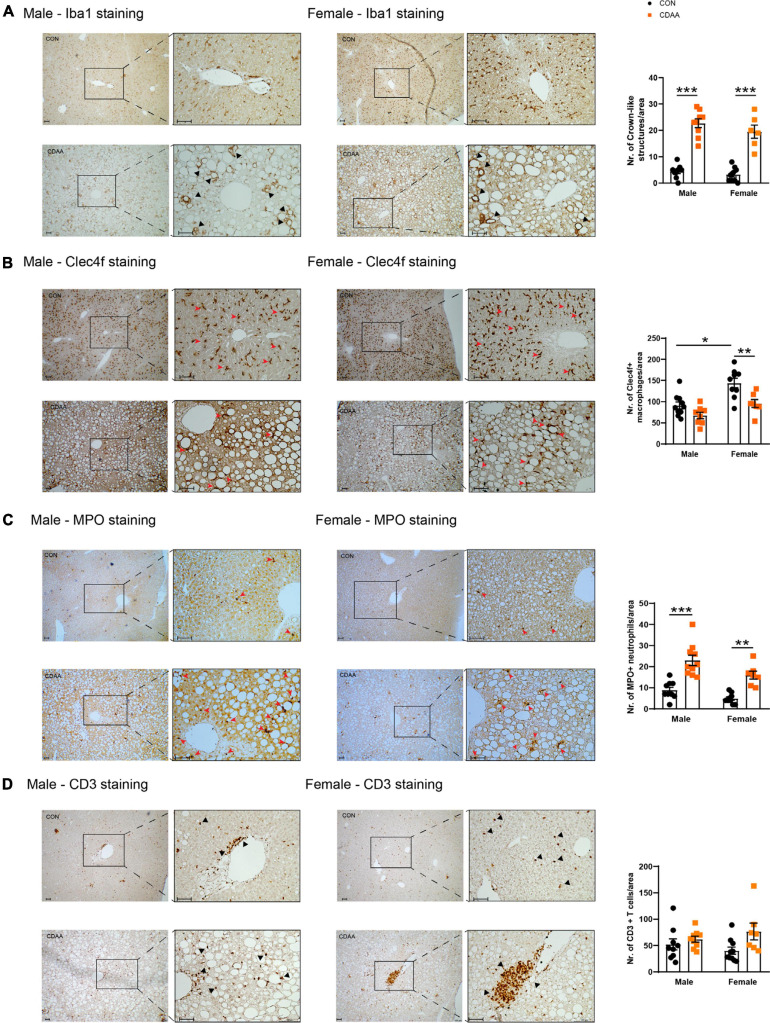
Hepatic inflammatory cell infiltrates in CDAA-induced NASH. The extent of liver inflammation was evaluated by immunohistochemical assessment and quantification of the presence of macrophages/monocytes on hepatic sections by Iba1 staining **(A)** and by the assessment and quantification of Clec4f positive resident Kupffer cells **(B)**. Infiltrating neutrophils are shown by MPO staining **(C)**. Average number of infiltrating neutrophil granulocytes on microscopic images were counted from hepatic histological sections **(C)**. Immunohistochemical assessment and quantification of the presence of T cells by CD3 staining **(D)**. Black and red arrows point to corresponding infiltrating cells. Scale bar indicates 100 μm. Results are shown as mean ± standard error of mean (SEM). *P < 0.05 was considered significant, **P < 0.01 was considered significant, ***P < 0.001 was considered significant.

The progression of NASH is closely related to the extent of hepatic damage, which is followed by reactive inflammation. In our model, a robust activation of the infiltrating monocytes/macrophages and a change in the number of Clec4f-positive resident macrophages a.k.a. Kupffer cells as well as the infiltration of neutrophils were detected (Figures 5A–C). Upon excessive intracellular cholesterol and fat accumulation, hepatocytes undergo necrosis and apoptosis. These necrotic cells are engulfed and encircled by macrophages forming the so-called crown-like structures. This histologic sign reflects robust inflammatory priming and activation which is likely to be related to the intracellular accumulation of cholesterol and cholesterol crystals in macrophages, and to the subsequent activation of inflammasomes (Ioannou, 2016).

We detected the presence of monocytes/macrophages with the pan-macrophage marker Iba1. Crown-like structures were detectable throughout the liver of CDAA diet-fed mice in both sexes, while in the control livers, Kupffer cells were localized next to the space of Disse. As a result of the CDAA diet, a similar increase in the number of crown-like structures was observed in both sexes (Figure 5A). In contrast, the Clec4f-positive resident Kupffer cells were detected in smaller numbers in female CDAA-fed animals. Once macrophages are activated, chemokines and cytokines are released to attract other inflammatory cell types, including neutrophils. We detected these infiltrating neutrophils by staining liver sections for myeloperoxidase (MPO), an enzyme involved in reactive oxygen species production. We saw a significantly increased number of MPO-positive neutrophils in both sexes of CDAA diet-fed mice (Figure 5C). The number of CD3-positive T cells, however, showed no change in any groups (Figure 5D).

**FIGURE 6:**
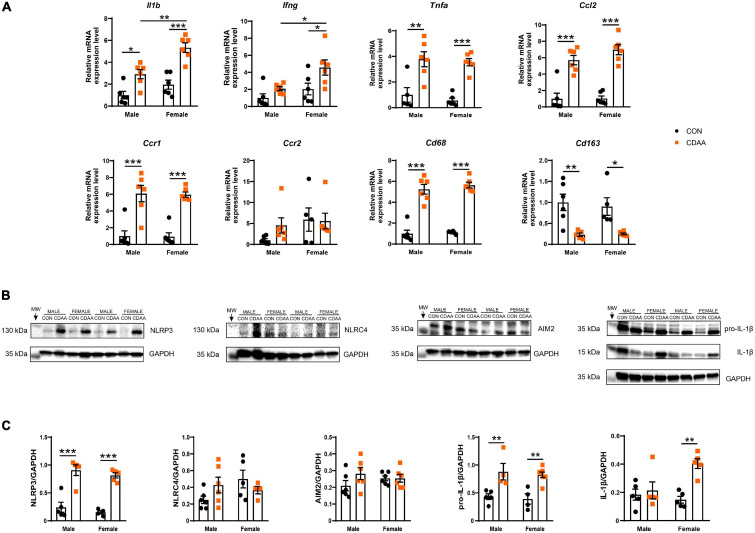
Hepatic inflammation in CDAA-induced NASH. Gene expression of pro-inflammatory (*Il1b, Ifng, Tnfa*), chemokine (*Ccl2, Ccr1, Ccr2*), and macrophage markers (*Cd68, Cd163*) genes were performed by qRT-PCR and was normalized to *Rpl13a*, as a housekeeping gene **(A)**. Representative Western blot analysis of inflammasomes sensors NLRP3, NLRC4, and AIM2, as well as pro-IL-1β and cleaved IL-1β **(B)**. Quantification of the Western blots **(C)**. Results are shown as mean ± standard error of mean (SEM). *P < 0.05 was considered significant, **P < 0.01 was considered significant, ***P < 0.001 was considered significant.

The activation of macrophages and infiltration of neutrophils was associated with a robust induction of the hepatic inflammatory gene expression profile. Certain characteristic markers of Th1-type immune activation such as *Il1b, Ifng*, and *Tnfa* showed upregulation due to CDAA diet feeding (Figure 6A). Out of these *Il1b* and *Ifng* showed significantly higher induction in female mice in comparison to male counterparts. *Ccl2*, a major chemoattractant, known to play a pivotal role in the progression of NASH, showed a robust induction due to dietary conditioning, while its receptor *Ccr2* showed no changes of expression. The chemokine receptor *Ccr1*, however, was induced by CDAA diet feeding.

The expression of IL-1β was further checked on the protein level. Pro-IL-1β was upregulated in both male and female CDAA-fed mice, however, cleaved IL-1β showed only an increase in female CDAA-fed mice, despite a similar degree of the expression of the NLRP3 inflammasome (Figures 6B,C).

Additionally, we tested the expression of monocyte/macrophage markers, to obtain a quantitative measure of their polarization state and to decide whether these changes were related to the activation of resident or infiltrating cells. We found a significant increase of the pan-macrophage/monocyte marker *Cd68* in the livers of CDAA-fed mice, which was paralleled by a reduction of the marker of resident, alternatively activated Kupffer cells (*Cd163*).

## Discussion

We report on the important morphological and molecular comparison of hepatic inflammatory and fibrotic changes as well as alterations in cholesterol metabolism-related proteins involving PCSK9 in middle-aged male and female mice upon NASH development in the CDAA diet-induced model. These relevant sex-specific differences are key in our understanding of disease development, and also in elucidating sex-specific responses to therapeutic interventions (Ventura-Clapier et al., 2017). Sex differences are widely accepted in various areas of clinical medicine, notably including cardiovascular and metabolic disorders, which are closely connected with NAFLD/NASH. However, preclinical studies, focusing on sex-specific changes are missing from the hepatology field (Lonardo and Suzuki, 2019).

The prevalence of NAFLD is higher in men than it is in women of reproductive age; however, following menopause, this sex difference is reduced or abolished (Gutierrez-Grobe et al., 2010), this age-dependent disparity between the sexes likely results from hormonal changes in women occurring as a result of menopause (Della Torre, 2020). In premenopausal women, hepatic metabolism and inflammation are under the control of sex hormones and serve reproductive needs (Della Torre et al., 2014). In addition, females tend to develop a stronger innate and adaptive immune response to antigens, which on one hand can accelerate pathogen clearance but on the other hand can lead to increased immune-related pathologies, such as autoimmune or inflammatory diseases (Beagley and Gockel, 2003). In Western societies, changes in the dietary and lifestyle habits of women, along with increased lifespan likely contributes to increased incidence of NASH and cardiometabolic diseases in the female population. Accordingly, it has been reported that more female patients die from NASH than men, suggesting that women may develop more severe or more rapidly progressing forms of the disease (Paik et al., 2019), however the mechanisms behind this phenomenon are not known.

Inflammation contributes to all stages of NASH development, from the initial steatosis to steatohepatitis, and final hepatocellular carcinogenesis (Tilg et al., 2016). Many studies have highlighted the prominent role of the NLRP3 inflammasome and cytokines of the interleukin-1 family in NAFLD and NASH (Thomas, 2017), thus inhibition of inflammasomes, or IL-1β signaling is an intriguing target in NASH (Kamari et al., 2011; Mridha et al., 2017; Tilg et al., 2020). Several studies strongly suggest that the key differences in the immune-inflammatory processes between sexes in cardiometabolic disease may be driven by sex hormones, stipulating that estrogen exerts anti-inflammatory effects, whereas testosterone primarily triggers inflammation (Fairweather, 2014). However, there is new evidence showing that the NLRP3 inflammasome plays a more prominent role in atherosclerosis in female mice than in males (Chen et al., 2020). These data further support our results, showing that during NASH middle-aged female mice have higher hepatic levels of IL-1β. These results offer an explanation for the sex-specific difference in the therapeutic responses to IL-1β inhibition in the CANTOS trial, where females tended to be more responsive than males to the IL-1β neutralizing antibody canakinumab (Ridker et al., 2017).

The discovery of PCSK9 as a circulating master regulator of LDLR has changed our understanding of cholesterol metabolism, which was traditionally considered to be tightly controlled by intracellular transcription factors (e.g., SREBPs) (Brown and Goldstein, 1997). Upon understanding the basic mechanism of PCSK9 action, exploitation of PCSK9 targeting therapies have emerged as safe and powerful tools to lower LDL-C levels and subsequent cardiovascular risk. New evidence suggests that NAFLD and NASH have strong multifaceted relationships with diabetes and metabolic syndrome, and are associated with increased risk of cardiovascular diseases (e.g., cardiac dysfunction, atherosclerosis, ischemic stroke), suggesting that the contribution of liver pathology may be independent of the presence of traditional CV risk factors. Therefore, therapies that could reduce the burden of NAFLD and NASH are of high clinical importance.

NASH development is a result of the complex interplay of pathologic changes in hepatocyte lipid metabolism, inflammatory signaling, and fibrosis. Although, the exact underlying mechanisms that directly induce inflammation and fibrosis are still unknown, several studies pointed out that perturbed cholesterol homeostasis is central to the development of NASH (Musso et al., 2013). Here, we show that adult female C57Bl/6J mice fed with a CDAA diet display reduced *Pcsk9* gene and protein expression in the liver and serum, which are associated with a more pronounced inflammatory phenotype. These results are somewhat surprising considering the well-described beneficial effects of PCSK9 inhibition on the overall lipoprotein metabolism, nevertheless, a similar change was reported by Lai et al. (2017) in a 5-weeks-long cholesterol-feeding challenge in mice. They identified the transcription factor E2F1 as a key regulator of hepatic PCSK9 expression, and speculated that E2F1-induced PCSK9 expression might be a key compensatory mechanism to avoid excessive cholesterol accumulation in hepatocytes. A similar conclusion was made by Lebeau et al. recently. They exposed PCSK9 knock-out mice to a high-fat diet and found that the genetic loss of PCSK9 led to a more severe form of steatohepatitis with more excessive lipid droplet formation, and augmented inflammatory gene expression and more pronounced fibrosis (Lebeau et al., 2019). Interestingly, they also found signs of insulin resistance in the PCSK9 knock-out animals.

Altogether these data suggest that PCSK9 deficiency (primarily genetic) could promote hepatic steatosis, at least in preclinical models with mice. Whether the pharmacological inhibition of PCSK9 either by monoclonal antibodies or siRNAs has similar effects in morbidly obese patients with NAFLD or NASH has not been studied yet, nor has the sex-specific hepatic response of patients to PCSK9 inhibitors. Hepatic steatosis has been identified as a safety concern during the development of some other cholesterol lowering drugs, such as lomitapide and mipomersen. Both of these drugs, by interfering with the assembly and secretion of (apoB)-containing lipoproteins, lead to hepatic steatosis in phase II and III clinical trials (Hooper et al., 2015; Panta et al., 2015).

## Conclusion

In conclusion, our study shows that sex-specific expression changes could be important mediators in hepatic cholesterol homeostasis, and in pro-inflammatory responses, suggesting that sex-specific therapeutic interventions might be more effective for the NASH indication. In our model, we found that the CDAA diet leads to the downregulation of hepatic *Pcsk9* expression which is associated with a pro-inflammatory phenotype seen in middle-aged female mice. Our study also highlights that there are important sex- and age-specific changes in cholesterol homeostasis, inflammatory response, and overall NASH development.

Limitations: Although here we investigated many novel aspects (age, sex) of NASH development, testing therapeutic interventions (e.g., PCSK9 inhibitors, IL-1β neutralizing antibodies), using knock-out animals, and following the time course of disease development was out of the scope of our study.

## Data Availability Statement

The raw data supporting the conclusions of this article will be made available by the authors, without undue reservation.

## Ethics Statement

The animal study was reviewed and approved by the National Scientific Ethical Committee on Animal Experimentation (PE/EA/1912-7/2017, Budapest, Hungary).

## Author Contributions

DK participated in study design and performed *in vivo* experiments, analyzed the data, and drafted the manuscript. VT, DG, IV, and ZO performed *in vitro* experiments and evaluated results. AG and PF revised the manuscript and the intellectual content and provided professional advice. ZV designed the experiments, wrote the manuscript, revised the intellectual content, and provided professional advice. All authors read and approved the final manuscript.

## Conflict of Interest

PF is the founder and CEO of Pharmahungary, a group of R&D companies. The remaining authors declare that the research was conducted in the absence of any commercial or financial relationships that could be construed as a potential conflict of interest.
