# Supplementary material for: Characterization of the CDAA Diet-Induced Non-alcoholic Steatohepatitis Model: Sex-Specific Differences in Inflammation, Fibrosis, and Cholesterol Metabolism in Middle-Aged Mice
Source: Front Physiol. 2021 Feb 22;12:609465. doi: 10.3389/fphys.2021.609465 (PMC7937716; doi:10.3389/fphys.2021.609465)
Supplement: Supplementary file 1 [file Table_1.docx]

**Gene name Accession number Forward primer Reverse primer Product size (bp)**

*Pcsk9* NM_153565.2 TTGCAGCAGCTGGGAACTT CCGACTGTGATGACCTCTGGA 76

*Ldlr* NM_010700.3   GAGGCTTGCGAGCCCAGATT ATGAGTCTTCTGCTGCAACTCCG 195

*Cd36* NM_001109218.1 CACAGGCTTTCCTTCTTTGC CCAGAACCCAGACAACCACT 106

*Ctgf* NM_010217.2 ATGTGGACCCCTCCTGATAGT TTCCAGTCGGTAGGCAGCTA 222

*Fn1* NM_010233.2 GAGTGCTGTGGTGGATTGGC GCCCAGTGATTTCAGCAAAGG 124

*Col1a1* NM_007742.4 TCTCCACTCTTCTAGTTCCT TTGGGTCATTTCCACATGC 226

*Col3a1*  NM_009930.2 GCTCGAGGCAATGATGGT ACCCTGCAGGTCCAACTTC 118

*Il1b*  NM_008361.4 GACAAAGCCAGAGTCCTTCAGAGAG CTAGGTTTGCCGAGTAGATCTC 147

*Ifng*  NM_008337.4 CGGCACAGTCATTGAAAGCC TGCATCCTTTTTCGCCTTGC 268

*Tnfa* NM_013693.3  TACTGAACTTCGGGGTGATTGGTCC CAGCCTTGTCCCTTGAAGAGAACC 295

*Ccl2* NM_011333.3 ACCTGCTGCTACTCATTCACC CTCTTGAGCTTGGTGACAAAAACTA 119

*Ccr1* NM_009912.4 ATACTCTGGAAACACAGACTCACT TTGCCCACCACTCCAATGAT 238

*Ccr2* NM_009915.2 AGGAGCCATACCTGTAAATGC GCCGTGGATGAACTGAGGTA 161

*Cd68* NM_001291058.1 TGCGGCTCCCTGTGTGT TCTTCCTCTGTTCCTTGGGCTAT 61

*Cd163* NM_001170395.1 GCCTCTGCTGTCACTAACGC AGATCCATCTGAGCAGGTCACT 354

*Rpl13a* NM_173340.2 GGATCCCTCCACCCTATGACA CTGGTACTTCCACCCGACCTC 131
